# Dynamics of The Expression of Pluripotency and Lineage Specific Genes in The Pre and Peri-Implantation Goat Embryo

**DOI:** 10.22074/cellj.2019.5732

**Published:** 2019-02-25

**Authors:** Pouria HosseinNia, Mehdi Hajian, Farnoosh Jafarpour, Seyed Morteza Hosseini, Mojtaba Tahmoorespur, Mohammad Hossein Nasr-Esfahani

**Affiliations:** 1 Department of Reproductive Biotechnology, Reproductive Biomedicine Research Center, Royan Institute for Biotechnology, ACECR, Isfahan, Iran; 2 Department of Animal Science, Faculty of Agriculture, Ferdowsi University of Mashhad, Mashhad, Iran; 3 Department of Research and Development, ROJETechnologies, Yazd, Iran

**Keywords:** Blastocyst, Embryo, Goat, Oocyte

## Abstract

**Objective:**

Two critical points of early development are the first and second lineage segregations, which are regulated by a wide spectrum of molecular and cellular factors. Gene regulatory networks, are one of the important components which handle inner cell mass (ICM) and trophectoderm (TE) fates and the pluripotency status across different mammalian species. Considering the importance of goats in agriculture and biotechnology, this study set out to investigate the dynamics of expression of the core pluripotency markers at the mRNA and protein levels.

**Materials and Methods:**

In this experimental study, the expression pattern of three pluripotency markers (*Oct4, Nanog* and *Sox2*) and the linage specific markers (*Rex1, Gata4* and *Cdx2*) were quantitatively assessed in in vitro matured (MII) oocytes and embryos at three distinctive stages: 8-16 cell stage, day-7 (D7) blastocysts and D14 blastocysts. Moreover, expression of Nanog, Oct4, Sox2 proteins, and their localization in the goat blastocyst was observed through immunocytochemistry.

**Results:**

Relative levels of mRNA transcripts for Nanog and Sox2 in D3 (8-16 cell) embryos were significantly higher than D7 blastocysts and mature oocytes, while *Oct4* was only significantly higher than D7 blastocysts. However, the expression pattern of *Rex1*, as an epiblast linage marker, decreased from the oocyte to the D14 stage. The expression pattern of *Gata4* and *Cdx2*, as extra embryonic linage markers, also showed a similar trend from oocyte to D3 while their expressions were up-regulated in D14 blastocysts.

**Conclusion:**

Reduction in *Nanog, Oct4, Sox2* mRNA transcription and a late increase in extra embryonic linage markers suggests that the developmental program of linage differentiation is retarded in goat embryos compared to previously reported data on mice and humans. This is likely related to late the implantation in goats.

## Introduction

A distinguishing feature of blastocyst formation in mammals is regulation of the trophectoderm (TE) and specification of the pluripotent inner cell mass (ICM) through a series of highly orchestrated events directed by spatial and temporal patterns of gene expression, cell polarization, and cell-cell interactions (1). The TE will differentiate into the placenta while the ICM differentiates into the epiblast and the hypoblast or primitive endoderm. Subsequently, the embryo proper is derived from the epiblast while extra-embryonic tissues are derived from the primitive endoderm and trophoblast. As the ICM of the newly developed blastocyst is the main source of embryonic stem cell (ESC) derivation in the mouse and human, it is obviously important to provide a clear understanding of the molecular circuitry governing ICM and TE ontogeny and to expand our knowledge of *in vitro* derivation of ESC and for their future applications in the goat species. 

Despite initial concepts proposing the equivalence of gene networks governing the delineation of ICM and TE and pluripotency across different mammalian species, recent comparative studies suggest that different pathways may be involved in controlling ICM-TE ontogeny in different species. For example, during first linage segregation, TE and ICM are committed and marked by reciprocal expression of *Cdx2* and *Oct4* in mouse blastocysts while derivation of the epiblast and primitive endoderm in second linage segregation is modulate by *Nanog* and *Gata6* , respectively (2). In humans, although a similar pattern of regulation exists, *OCT4* is not restricted to the ICM and it has been demonstrated that in primates ESCs and isolated ICMs fail to incorporate into host embryos and develop into chimeras (3). More importantly, it has been recently shown that primate ESCs are more equivalent to mouse epiblast stem cells (EpiSCs), which are driven from post implantation embryos and are developmentally more advanced relative to naive ESCs (4). 

Ungulates may be a unique case, having some similar regulatory pathways to mouse and human cells but is coupled with dramatically distinct expression patterns. For example, comparative immunocytochemical studies have shown that *Cdx2* and *Gata6* expression in porcine and bovine blastocysts resembled that of the mouse, however, Oct4 is expressed in both the ICM and TE (5). Importantly, through exchanging mouse and bovine Oct4 reporters, Berg et al. (6) elegantly demonstrated that the mouse Oct4 promoter, which is normally repressed in the mouse TE remained active in the bovine TE; and vice versa, while bovine *Oct4* promoter also remains active in the mouse TE, suggesting that the TE is not committed at an equivalent stage in the bovine embryo as it is in newly developed mouse blastocysts. In this regard, a recent study by Simmet et al. (7) showed that *Oct4* is expressed during early stages of embryonic development (oocyte to morula stage) and regulates *Nanog, Gata6* and *Gata4* expression in bovine embryos as it does in the mouse (8), however, unlike in the mouse this is not mediated through fibroblast growth factors (FGF) signaling. 

The great difference between ICM and TE cells, commonly occurs within two cell cycles from morula to the blastocyst (9). A growing body of evidence indicating that the core pluripotency triad in humans (*OCT4, NANOG, SOX2*) and mice (*Oct4, Nanog, Sox2*) is the main regulator of the establishment and maintenance of pluripotency in the ICM. The expression levels of the core pluripotency triad during ICM emergence in mice and humans have been well established at the mRNA and protein levels. However, the actual status of *Oct4, Nanog,* and *Sox2* genes is poorly understood in other mammals. Such studies will provide a roadmap for differentiating definitive species-specific differences and help to understand why authentic ESCs are not established in ungulates (4, 7). 

The goat is a valuable livestock with promising importance in agriculture, biomedicine and transgenic production of pharmaceutical drugs. Therefore, this study set out to investigate the dynamics of the expression of the core pluripotency triad in *in vitro* produced goat embryos at the mRNA and protein levels. Moreover, since implantation in ungulates, unlike in human and mouse embryos, occurs with a delay of around 7 days, this period of "delay" in implantation should likely "influence" the pattern of developmentally important genes (10). Therefore, we further planned to evaluate the expression status of peri-implantation goat embryos cultured in vitro until D14.

## Materials and Methods

Unless otherwise stated, all chemicals and media were obtained from Sigma Chemical Co. (St. Louis, MO, USA) and Gibco (Grand Island, NY, USA), respectively.

### Selection of the gene set

In order to select the genes that could predominantly be involved in the regulation of early embryonic development and pluripotency, and due to a lack of sufficient data on the goat species, we followed the strategy used by McGraw et al. (11). In brief, we sought the related information using gene expression databases that profile gene expression and gene ontologies (GOs) in human and mouse embryos and ESCs. To be a candidate, the potential genes had to be commonly present in ESCs and either in the oocyte or the blastocyst, while playing a critical role in transcription regulation and pluripotency. This survey provided a list of 6 genes including, *Oct4, Rex1, Sox2, Nanog, Gata4, Cdx2* genes.

### *In vitro* production of goat embryos

The procedure for *in vitro* production of goat embryos was as has been described previously (12). In brief, goat ovaries were used for *in vitro* maturation of cumulus-oocyte complexes (COCs) in tissue culture medium-199 (TCM199) plus 10% fetal calf serum (FCS), 2.5 mM sodium pyruvate, 100 IU/mL penicillin, 100 mg/mL streptomycin, 10 mg/mL follicle stimulating hormone (FSH), 10 mg/mL luteinizing hormone (LH), 1 mg/mL estradiol-17β, and 0.1 mM cysteamine under mineral oil for 20-22 hours at 39˚C, 5% CO_2_, and maximum humidity before being used for embryo development in groups of six in 20 μl droplets of a modified formulation of synthetic oviductal fluid (mSOF) (13) at 39˚C, 6% CO_2_, 5% O_2_, and maximum humidity. MII oocytes were collected 20-22 hours post maturation, D3 developing embryos at the 8-16 cell stage, and D7 blastocysts, were washed thrice in phosphate buffered saline (PBS) without calcium and magnesium, and collected. Pools of 60 oocytes, 35-40 day 3 embryos, 20 day 7 blastocysts were collected in 500 μL microtubes containing 20 μL RLT buffer, frozen and stored at -70˚C until RNA extraction. All oocyte and embryo pools used for RNA extractions were collected and analyzed in triplicates. This system of embryo development supported quite good rates of *in vitro* embryo development with cleavage and blastocyst rates ranging between 85-92% and 40-45%, respectively (14). In order to extend *in vitro* culture of goat blastocysts, we prepared a feeder layer of caprine fetal fibroblasts (CFFs) as described by Behboodi et al. (10). For this purpose, a CFF line was derived from three 40-day male fetuses. Single cell suspension was prepared by mincing fetal tissue and culturing the cells in Dulbecco’s modified eagle medium and ham’s F12 (DMEM/F12) supplemented with 10% FBS, 0.25 % amphotericin-B, 1% penicillin-streptomycin, 1% gentamycin in 25 cm^2^ culture flasks and incubated at 37˚C, 6% CO_2_, until the appearance of a confluent monolayer from day 4 onwards. The monolayer was trypsinized and further cultured for proliferation of the CFF source, each passage took around 3-4 days until becoming confluent. CFFs at passages 2-4 were treated with mitomycin (10 mg/mL) for 2 hours. Mitomycin-treated cells were washed twice with DMEM/F12, and treated with 0.25% trypsin-EDTA and dissociated into single cells by gentle pipetting. Cells were then seeded at concentration of 1×10^5^ cells/mL in 100 μL drops of DMEM/F12 in the vicinity of feeder-free 100 μL droplets of DMEM/F12 supplemented with 10% FBS, 1% L-glutamine, 1% non-essential amino acid, and 0.1% β-mercaptoethanol under mineral oil. Five to six D7 blastocysts were transferred to each 100 μL droplet of feeder-free DMEM/F12. Using of the tip of a drown glass pipette, the DMEM/F12 drops containing blastocysts were gently connected to their adjacent DMEM/F12 drop containing the CFF monolayer. This joined culture system provides the beneficial effects of a feeder layer for extended *in vitro* embryo culture, while preventing attachment and flattening of the growing blastocysts. The joined droplets were refreshed every other day until D14 of embryo development, when pools of 7-10 well developed spherical D14 embryos were pooled for RNA extraction as described above.

### RNA extraction and reverse transcription polymerase chain reaction 

The procedure for quantitative real-time polymerase chain reaction (qRT-PCR) was as described previously (15). In brief, total RNA of MII-oocytes, 8-16 cell embryos, blastocysts on days 7 & 14 was extracted using RNeasy Micro kit (Qiagen, ON, Canada) followed by the treatment with DNase I (Ambion, ON, Canada) according to the manufacturer’s protocol. The quality and quantity of the extracted RNA was determined using a WPA Biowave spectrophotometer (Cambridge, UK). For reverse transcription, 10 µL of total RNA was used in a reaction with a final volume of 20 µL containing 1 µL of Random Hexamers, 4 µl RT buffer (10 x), 2 µL of dNTP, 1µl of RNase inhibitor (20 IU), and 1µl of reverse transcriptase (Fermentas, Glen Burnie, Ontario, Canada). Reverse transcription was carried out at 25°C for 10 minute, 42°C for 1hour and 70°C for 10 minutes. 

### Quantitative analysis of transcripts by real time- polymerase chain reaction 

The transcript level of the aforementioned genes and ACTB, as a housekeeping gene, were measured using real time-PCR (RT-PCR). Briefly, total RNA of oocytes, day3 embryos, day 7 and 14 blastocysts was extracted and then each RNA sample was used for cDNA synthesis. RT-PCR was carried out using 1 µL of cDNA (50 ng), 5 µl of the SYBR Green/0.2 µl ROX qPCR Master Mix (2X) (Fermentas, Germany) and 1 µL of forward and reverse primers (5 pM) adjusted to a total volume of 10 µL using nuclease-free water. The primer sequences, annealing temperatures and size of the amplified products are shown in Table 1. 

### Embryo immunostaining

Expression of Nanog, Oct4 and Sox2 proteins and their localization in the goat blastocyst was observed through immunocytochemistry (ICC). *In vitro*-derived embryos were washed in PBS containing 1 mg/ml polyvinyl alcohol (PVA), and then fixed in 4.0% paraformaldehyde for 30 minutes. Subsequently the embryos were washed in PBS/PVAwith 0.5 µl/ml tween 20 (solution1). Permeablization was carried out in 0.5% Triton X-100 (Sigma-Aldrich) solution in PBS for 15 minutes at room temperature (RT), and then washed with solution1. In order to block non-specific binding sites, embryos were incubated in blocking solution containing PBS/PVA containing 1% bovine serum albumin (BSA)+10% normal goat serum for 60 minutes at RT. Subsequently, embryos were incubated with the primary antibody, either rabbit polyclonal antibody against Nanog (1:300 dilution, Abcame, ab21603), rabbit monoclonal anti-human Sox2 antibody (1:300 dilution, cell signaling, 3579) and rabbit polyclonal anti-mouse Oct4 (1:300 dilution, lifespan, c48532), for 60 minutes at 37°C. Then, embryos were washed 3-4 times in PBS/PVA for 15 minutes at 37°C and subsequently incubated in goat anti- rabbit IgG fluorescein conjugated (1:50 dilution, Sigma, F1262) for 45 minutes at RT. After washing 3-4 times in PBS/ PVA at 37°C, all embryos were counterstained with 1 µg/mL Hoechst for 5-10 minute and then washed 3-4 times in PBS/ PVA for 15 minute at 37°C, Embryos were mounted in 10ml light diagnostics mounting fluid (Merck, Germany) on a slide before observation. Fluorescent signals were visualized using a fluorescent microscope (Olympus, Japan). 

**Table 1:** Specific real-time primers were designed for gene sequences


Gene	Primer sequences (5ˊ-3ˊ)	Length of PCR product	Tm

*OCT4*	F: GCCAGAAGGGCAAACGAT	96	56
	R: GAGGAAAGGATACGGGTC		
*REX1*	F: GCAGCGAGCCCTACACAC	94	61
	R: ACAACAGCGTCATCGTCCG		
*SOX2*	F: ATGGGCTCGGTGGTGA	182	54
	R: CTCTGGTAGTGCTGGGA		
*NANOG*	F: GATTCTTCCACAAGCCCT	137	54
	R: TCATTGAGCACACACAGC		
*GATA4*	F: TCCCCTTCGGGCTCAGTGC	128	64
	R: GTTGCCAGGTAGCGAGTTTGC		
*CDX2*	F: CCCCAAGTGAAAACCAG	144	53
	R: TGAGAGCCCCAGTGTG		
*ACTB*	F: CCATCGGCAATGAGCGGT	146	60
	R: CGTGTTGGCGTAGAGGTC		


PCR; Polymerase chain reaction and Tm; Melting temperature.

### Statistical analysis

Statistical significance was considered to be P<0.05 and determined by two-tailed Fisher’s exact test in SPSS software version 20 for developmental data, two-tailed student’s t test with equal variance for cell counts and real-time PCR data was used.

## Results

### Gene expression pattern

**Fig.1:**
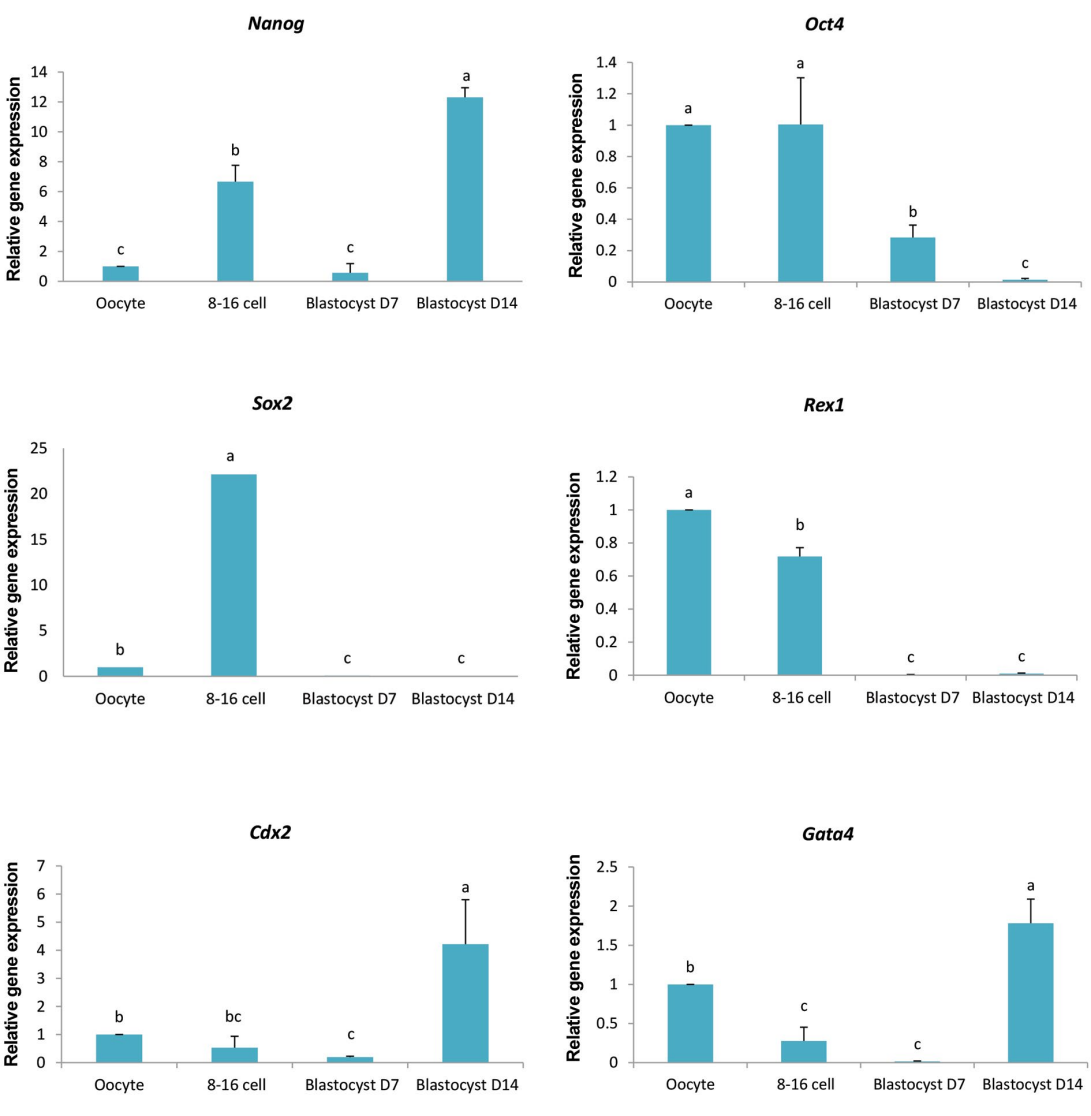
Relative gene expression of specific lineage markers for the ICM, TE, or PE in goat oocytes and preimplantation embryos. a, b, c symbols showed significant differences between the developmental stages. Error bars represent standard deviation. ICM; Inner cell mass, TE; Trophectoderm, and PE; Primitive endoderm.

In order to understand the relation between the stages of embryonic development and linage segregation properties, we investigated expression of several pluripotency-related genes (*Oct4, Sox2* and *Nanog*), a lineage specific marker for TE development (*Cdx2*), as well as markers for the development of the primitive endoderm and the ICM (*Gata4* and *Rex1*, respectively) at various embryonic development stages. Oocytes, day 3 embryo (D3), day 7 (D7) and 14 (D14) blastocysts were collected and mRNA transcript levels were determined by RT-PCR CT-values for the aforementioned markers (Fig .1). 

In the case of *Nanog*, the relative expression levels of mRNA transcripts in day 3 embryos and D14 blastocysts were significantly higher than oocytes and D7 blastocysts. The expression of *Sox2* was relatively low in the oocytes and significantly increased by day 3 embryos and subsequently decreased to significantly lower values compared to oocytes. The pattern of expression for *Oct4* was not significantly lower in D3 embryos compared to oocyte but it significantly decreased by D7 and D14 compared to D3 embryos. 

*Rex1* expression was similar to that of Oct4 and its expression was significantly higher in oocytes compared to D3 embryos and it significantly decreased in D7 and D14 blastocysts compared to oocytes and D3 embryos. *Cdx2* mRNA was detected between oocytes and D14 blastocyst, but its expression was meaningfully up-regulated in D14 blastocysts, when compared with previous stages. The expression pattern of the lineage marker *Gata4* was highest in D14 blastocysts, when compared to earlier stages. *Gata4* expression gradually decreased from oocytes to D7 blastocysts and became significantly elevated by D14.

**Fig.2:**
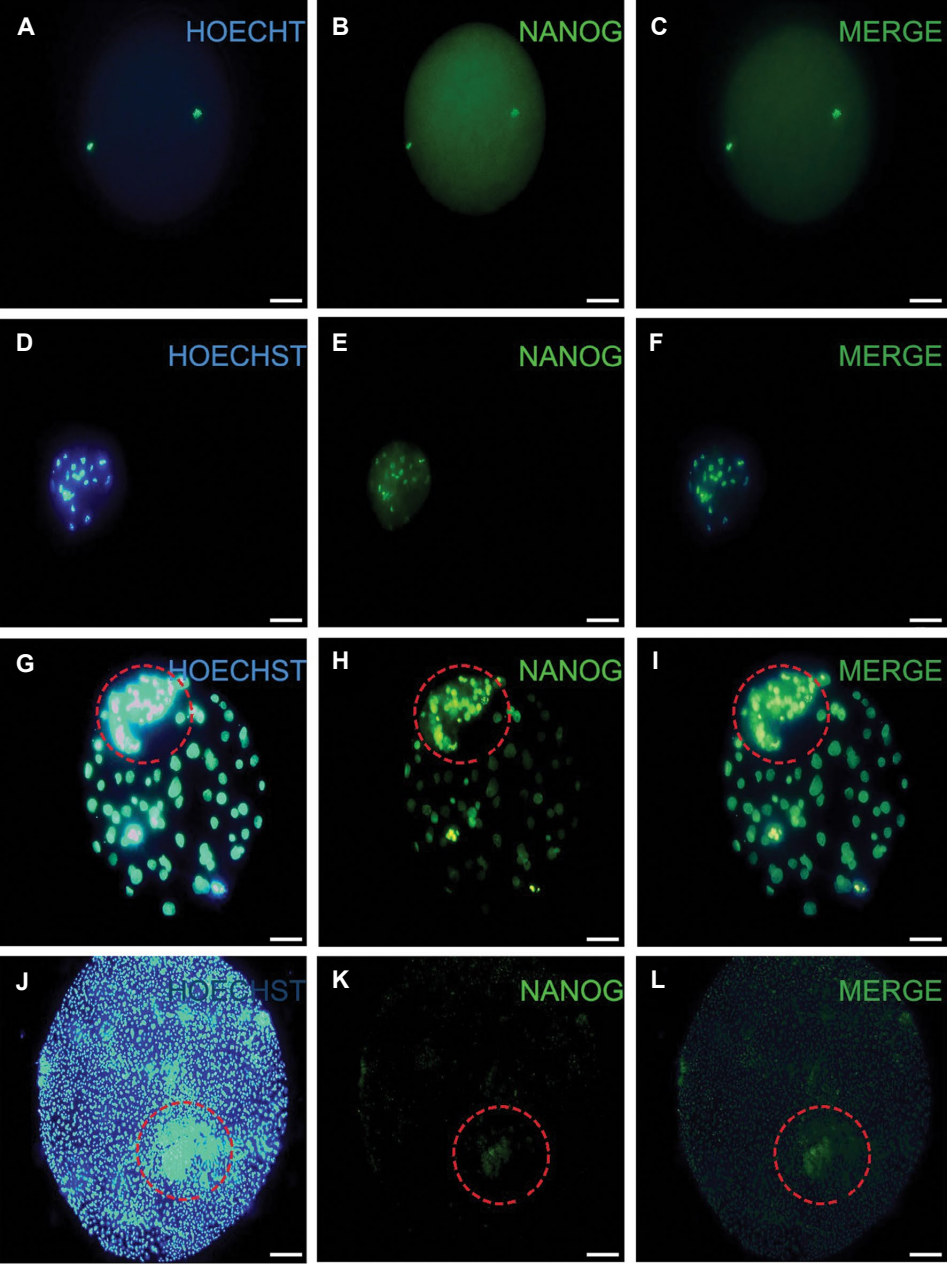
Nanog immunofluorescence results for in vitro-produced goat oocyte, 8-16 cell stage, blastocyst day at 7 stage, blastocyst day at 14 developmental stage. A-C. Staining of nuclear and embryo cells with HOECHT, Nanog antibody and merge respectively in oocyte stage, D-F. Staining of embryo cell in 8-16 cell stage in the above manner, G-I. Staining of embryo cells in blastocyst at day 7 stage in the above manner, and J-L. Staining of embryo cells in blastocyst at day 14 stage in the above manner. Dashed line denotes inner cell mass (ICM) (scale bar: 200 µM).

### Immunostaining results

Nanog, Oct4 and Sox2 protein expression and localization in goat blastocysts were observed using ICC. Since, the ICM in the goat blastocyst is not very clear or distinguishable, whole immunostaining was used to examine the expression and localization of factors associated with lineage segregation. Nanog expression was detectable in goat oocytes, D3 embryos, D7 and D14 blastocysts. Expression of Nanog appeared tobe localized in the nuclei and nucleoplasm of ICM cells andit appeared to be restricted to the nuclei of TE cells. In D7blastocysts, the fluorescent intensity of Nanog in the ICMappeared to be higher than in the TE, but in D14 blastocysts, Nanog was expressed exclusively in the ICM (Fig .2). 

**Fig.3:**
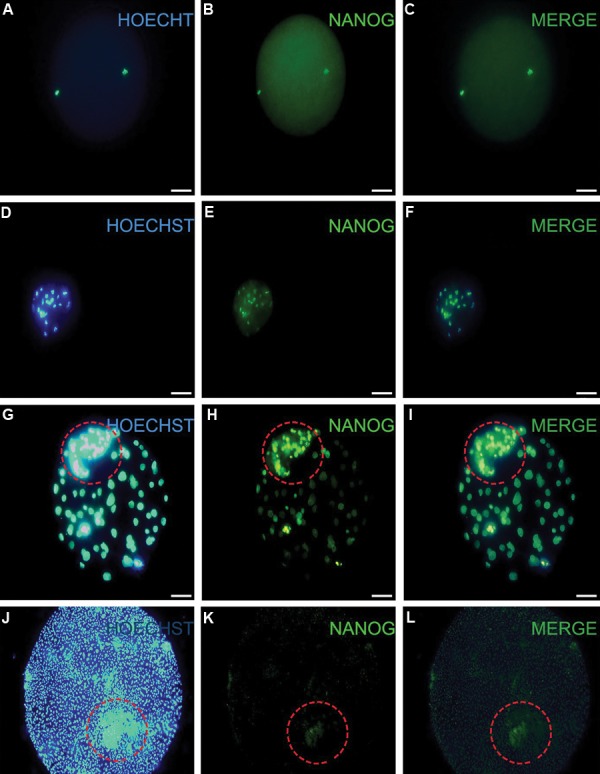
Oct4 immunofluorescence results for in vitro-produced goat oocyte, 8-16 cell stage, blastocyst day at 7 stage, blastocyst day at 14 developmental stage. A-C. Staining of nuclear and embryo cells by HOECHT, Oct4 antibody and merge respectively in oocyte stage, D-F. Staining of embryo cell in 8-16 cell stage in the above manner, G-I. Staining of embryo cell in blastocyst at day 7 stage in the above manner, and J-L. Staining of embryo cells in blastocyst at day 14 stage in the above manner. Dashed line denotes inner cell mass (ICM) (scale bar: 200 µM).

**Fig.4:**
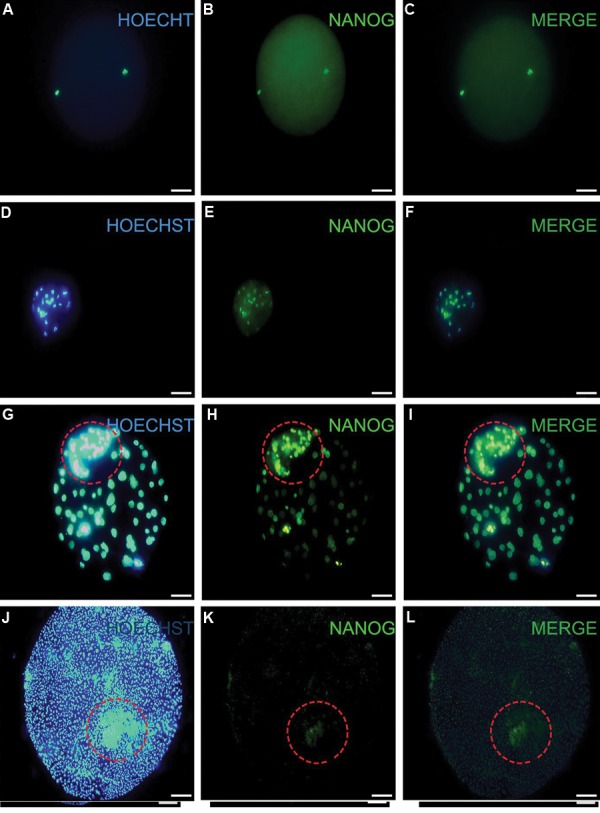
Sox2 immunofluorescence results for in vitro-produced goat oocyte, 8-16 cell stage, blastocyst day at 7 stage, blastocyst day at 14 developmental stage. **A-C.** Staining of nuclear and embryo cells by HOECHT, Sox2 antibody and merge respectively in oocyte stage, **D-F.** Staining of embryo cell in 8-16 cell stage in the above manner, **G-I.** Staining of embryo cell in blastocyst at day 7 stage in the above manner, and **J-L.** Stain of embryo cell in blastocyst at day 14 stage in the above manner. Dashed line denotes inner cell mass (ICM) (scale bar: 200 µM).

**Fig.5:**
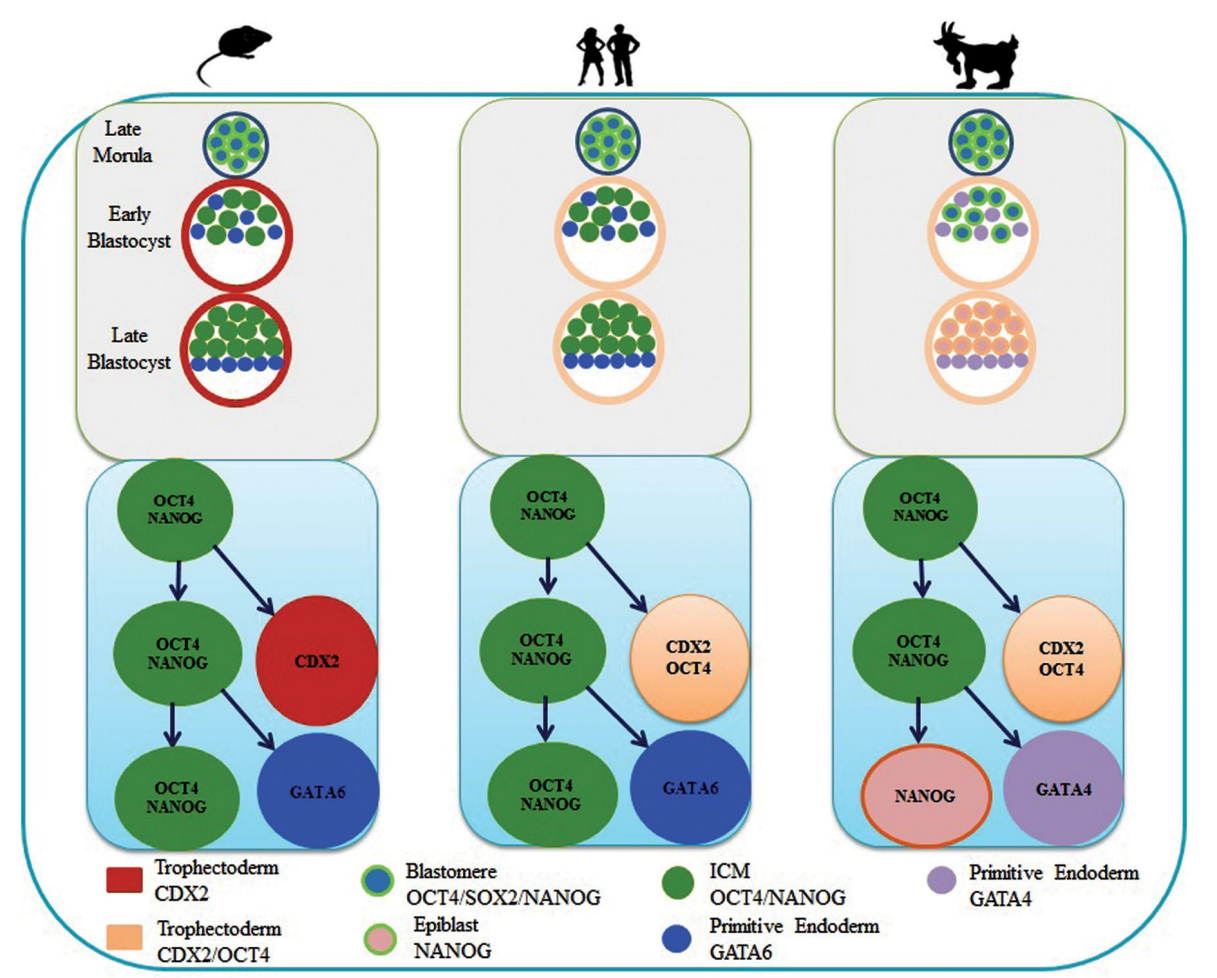
Early lineage segregation in mouse, human, and goat. Oct4, Nanog and Sox2 have been expressed in a different manner in goat embryos compared to mouse or human embryos, where these factors play a role in the formation of the pluripotent primitive ectoderm.

Oct4 expression was detected from the oocyte to Sox2 protein expression was also limited to ICM cells the D14 blastocyst stage. Its expression appeared to especially in blastocysts on D14, however in D7 goat be restricted to the nuclear area but it was difficult to blastocyst also appeared to be expressing it in the TE discern its distribution between ICM and TE (Fig .3). (Fig .4, 5). 

## Discussion

Most of the information that we have about the development and genetics of the embryo is derived from studies carried out on mouse and human embryos. These studies mark two fundamental stages of linage segregation. The first one is the distinction of TE from ICM, which occurs after a reciprocal constraining of *Oct4* and *Cdx2* (2, 16) and the second lineage segregation, which occurs as a result of the mosaic expression of Nanog and Gata6 which occurs in the ICM and causes the separation of the primitive ectoderm and primitive endoderm (17). To assess the same concept in goats, we also assessed the expression of the core pluripotency triad (*Oct4, Nanog* and *Sox2*) at both RNA and protein levels and the expression of linage markers (*Rex1, Gata4* and *Cdx2*) during goat pre-implantation embryo development. 

*Nanog* mRNA was presented in goat oocytes and has two waves of expression, peaking at around the 8 cell stage (D3), and D14, while being low in D7 blastocysts. Localization assessment of *Nanog* revealed its expression is similar between different blastomeres and appears to be present mainly in the nucleus but by D7, a salt and pepper appearance is observed in the ICM as in other species (18). This is likely due to lineage-specific markers *Gata6* and *Nanog*. Unlike in the mouse it is expressed in the nucleus of trophoblast cells and finally becomes restricted to the ICM by D14. The "salt and pepper" appearance of Nanog in the ICM, as in other species, may reflect its differentiation to epiblast and hypoblast or primitive endoderm. FGF4 appears to be the main mediator of this segregation in mouse embryos and lack of FGF4 results in *Nanog* enrichment but in bovine embryos as an ungulate this effect is not mediated through FGF and in the goat it remains to be defined. Expression of Nanog protein in the nucleus of trophoblast cells may be related to proliferation of the trophoblast known as embryo elongation which occurs before embryo implantation during D7-14 post fertilization in goats (10). 

The first peak in the expression of *Nanog* may be related to embryonic genome activation, which is required for the maintenance of pluripotent cells for early gastrulation, as Nanog is also considered as a pluripotent lineage specific marker in bovine cells (19). The second peak may be related to the increased number of epiblast cells required by embryos to undergo the process of gastrulation. It is interesting to note that, unlike in the mouse and human, in most ungulates, *Nanog* decreases during transition from D3 to D7 (20) but as stated, presence of the protein in the nucleus of trophoblast cells may be related to embryo elongation. Indeed, in this regard, it has been shown that *Nanog-/-* cells expand more slowly than wild- type cells (21) and that *Nanog* plays a role in proliferation of cancer cells (22) and can also increase proliferation in somatic cells (23). 

The reduction in expression of Nanog from day 3 to 7 is very likely related to the time of implantation and gastrulation between these species. Indeed, Sun et al. (24) have stated that the second peak of Nanog mRNA expression (D14) is associated with the increased number of epiblast cells, as it has been shown in mice, that Nanog through Nodal/Smad2 signaling leads to consolidation of epiblast pluripotency. *Nanog* is also a prerequisite for the formation of the primitive endoderm through an independent mechanism (25).

Unlike *Nanog,* the expression of *Oct4* in goats gradually decreases from oocyte through to day 14. In this species *Oct 4* is expressed in all the nuclei of the morula-stage embryos. By blastocyst stage a differential expression of *Oct4* is observed but it is not completely extinguished as cells where very rarely found to be *Oct4* positive in day 14 blastocysts. Indeed, high *Oct4* levels in the oocyte is likely to be related to the acquisition of meiotic competence (26) as it has been stated ” that a primary role of Oct4 at the initiation of genome activation may be more related to maintenance rather than transcriptional regulation required for the initial establishment of the inner-cell mass. In mice expression of *Oct4* in the oocyte does not appear be essential until later in development, i.e. formation of the PE and when the expression of multiple EPI and PE genes such as *Gata6* and FGF4 are required, but exploration of this issue in other species reveals a different story. In both human and bovine development, *Oct4* appears to be essential for first linage differentiation and thereby blastocyst formation (7). The presence of *Oct4* in all the nuclei in the morula stage is consistent with the pattern of *Oct4* expression in other species. Its differential expression in day 7 blastocysts is consistent with observations in human and bovine embryos but is in contrast to the mouse where expression of Oct4 becomes non existant in TE cells which has been attributed to the speedy differentiation of the TE required for implantation of the embryo. In ungulates, *Cdx2* and Oct4 are co- expressed in the TE until the time of implantation (14) and reciprocal expression of *Cdx2* and *Oct4* in goats by D14 may suggest that a similar trend is taking place except for the fact that this trend is delayed by 7 day required for the elongation of the embryo which is mainly mediated through the expansion of TE cells. 

Assessment of the relative expression of *Sox2* revealed that its low expression in the oocyte and increased expression around day3 coincides with the time of maternal embryonic transition. Differential expression of *Sox2* by different cells of the embryo is apparent on day3 and gradually becomes restricted to the ICM by day14. Indeed, in mice, it has been reported that a limited level of Sox2 expression is required to allow development past the morula (27). Moreover, *Sox2* has been considered as the main "driver of the earliest heterogeneity within the ICM, a heterogeneity that leads to the EPI/PE cell fate decision" based on Sox2 concentration (28). *Sox2*, despite being an Oct4 binding partner, its expression in bovine embryos appears to be independent of *Oct4*, as the absence of Oct4 does not prevent the expression of *Sox2* (7), despite embryos arresting at the morula stage (29). In addition, Sox2 appears to be essential for formation of TE cells in mice (28). Detection of Sox2 through immunostaing and gradual reduction in expression of *Sox2* mRNA by D14 may suggest that the remaining mRNA might be stable and may account for its protein expression observed in the ICM in day 14. A second possibility for the decrease in relative expression of Sox2 mRNA by day 7 and 14, and detection of its protein by means of immunostaing may also be related to the skewed ratio of expression of Sox2 in the ICM relative to TE cells, but this possibility needs further exploration. 

Based on cell tracing studies, *Cdx2* is considered as the main regulator of the TE lineage in mice and many other species including bovine and porcine embryos (3032). In bovine embryos, the expression of *Cdx2* is also high in TE relative to ICM (18), unlike in the mouse, which is considerably low in the ICM. In the goat, the increased expression of *Cdx2* and decreased expression of *Oct4* on day14 may suggest that, similar to the mouse, the regulation of Cdx2 is also controlled by decreased expression of Oct4. But this is an associative effect, which needs further verification in this species. Increase in expression of Gata4 on day 14, as the marker of the primitive endoderm, also supports the possibility of an inverse relation between *Oct4* with *Cdx2* and Gata4, but as stated, it needs further verification. It is of interest to note that, as in the mouse, decreased expression of *Oct4* in the goat is also concomitant with the formation of embryonic layers on day 14. *Rex1* plays an important role in maintaining pluripotency (33) in goats and the decreased expression of *Rex1* is likely to be due to the outstanding increase in the rate of TE proliferation compared to ICM cells, which is very likely to be related to embryo elongation in this species. 

This study has a few shortcomings which need to be considered in future studies. These included: i. The antibodies used are not specific to goat, ii. The ICM and TE need to be separated to discern the differential expression of these markers, and iii. The role of each gene in development needs to be assessed in knockout and knockdown studies. 

## Conclusion

In this study for the first time we assessed the triad of pluripotency genes and lineage specific markers at the mRNA level and we used immunostaining to assess pluripotency markers. Overall, the pattern of expression for the triad markers and their restriction between ICM and TE in the goat is similar to previous reports in the mouse and human. However, the pattern of expression of linage specific markers appears to be delayed in D7 blastocysts. This difference appears to be due to delayed implantation in ungulates.
